# Fucoidan Inhibits the Proliferation of Human Urinary Bladder Cancer T24 Cells by Blocking Cell Cycle Progression and Inducing Apoptosis

**DOI:** 10.3390/molecules19055981

**Published:** 2014-05-09

**Authors:** Hye Young Park, Gi-Young Kim, Sung-Kwon Moon, Wun Jae Kim, Young Hyun Yoo, Yung Hyun Choi

**Affiliations:** 1 Department of Pharmacy, Busan National University, Busan 609-735, Korea; E-Mail: nattier2895@naver.com; 2 Department of Marine Life Sciences, Jeju National University, Jeju 690-756, Korea; E-Mail: immunkim@cheju.ac.kr; 3 School of Food Science and Technology, Chung-Ang University, Ansung 456-756, Korea; E-Mail: sumoon66@dreamwiz.com; 4 Department of Urology, College of Medicine, Chungbuk National University, Cheongju 361-763, Korea; E-Mail: wjkim@chungbuk.ac.kr; 5 Department of Anatomy and Cell Biology, Dong-A University College of Medicine and Mitochondria Hub Regulation Center, Busan 602-714, Korea; 6 Department of Biochemistry, Dongeui University College of Korean Medicine, Busan 614-052, Korea; 7 Anti-Aging Research Center & Blue-Bio Industry RIC, Dongeui University, Busan 614-714, Korea

**Keywords:** fucoidan, bladder cancer, G1 arrest, apoptosis, p21, caspase, tBid

## Abstract

Although fucoidan has been shown to exert anticancer activity against several types of cancer cell lines, no reports have explored fucoidan-affected cell growth in human urinary bladder cancer cells. In this study, we investigated the anti-proliferative effects of fucoidan in human bladder cancer T24 cells. Our results indicated that fucoidan decreased the viability of T24 cells through the induction of G1 arrest and apoptosis. Fucoidan-induced G1 arrest is associated with the enhanced expression of the Cdk inhibitor p21WAF1/CIP1 and dephosphorylation of the pRB along with enhanced binding of p21 to Cdk4/6 as well as pRB to the transcription factor E2Fs. Further investigations showed the loss of mitochondrial membrane potential and the release of cytochrome *c* from mitochondria to cytosol, proving mitochondrial dysfunction upon fucoidan treatment with a corresponding increase in the Bax/Bcl-2 expression ratio. Fucoidan-triggered apoptosis was also accompanied by the up-regulation of Fas and truncated Bid as well as the sequential activation of caspase-8. Furthermore, a significant increased activation of caspase-9/-3 was detected in response to fucoidan treatment with the decreased expression of IAPs and degradation of PARP, whereas a pan-caspase inhibitor significantly suppressed apoptosis and rescued the cell viability reduction. In conclusion, these observations suggest that fucoidan attenuates G1-S phase cell cycle progression and serves as an important mediator of crosstalk between caspase-dependent intrinsic and extrinsic apoptotic pathways in T24 cells.

## 1. Introduction

Both cell cycle and apoptosis control mechanisms serve major regulatory functions of growth and development in all living organisms. Therefore, cell cycle deregulation and apoptosis resulting in uncontrolled cell proliferation are the most frequent alterations that occur during the development and progression of cancer. For this reason, a blockade of the cell cycle and apoptosis induction are regarded as effective strategies for eliminating cancer [1,2]. In mammalian cell cycle regulation, key regulator proteins are cyclin-dependent kinases (Cdks), whose activity is specifically controlled by cyclins and Cdk inhibitors at specific points of the cell cycle [3,4]. In particular, the G1 checkpoint is the most conspicuous target for many anticancer agents. D-type cyclins, cyclin E and Cdk4/6, Cdk inhibitors, including p21 and p27, and retinoblastoma protein (pRB) are the central players of G1 phase transition [5,6]. Apoptosis is a mechanism responsible for maintaining tissue homeostasis by mediating the equilibrium between cell proliferation and death. The key mediators of apoptosis are caspases, a family of cysteine proteases that cleave a critical set of cellular proteins near specific aspartic acid residues [7,8]. Caspase-dependent apoptosis occurs *via* two separate yet interlinked signaling mechanisms: the extrinsic death receptor-mediated pathway triggered by the activation of death receptors leading to the activation of caspase-8, and the intrinsic mitochondria-mediated pathway initiated by the release of cytochrome *c* from the mitochondrial matrix following the loss of inner mitochondrial membrane integrity and activation of caspase-9 [9,10,11,12]. Therefore, the induction of cell cycle arrest associated with apoptotic cell death is one of the strategies for anticancer drug development.

Among natural sources, marine organisms are a novel and rich source of bioactive compounds. Algae and seaweeds in particular have great potential as supplements in functional foods or for the extraction of compounds, and they have been used an important healthcare medicinal foods and pharmaceutical agents in Asian communities [13,14,15]. They are known for their richness in polysaccharides, minerals, and certain vitamins, but they also contain bioactive substances like proteins, lipids, and polyphenols. Fucoidan is a naturally occurring polysaccharide isolated from various species of brown algae and brown seaweed. This compound contains considerable amounts of L-fucose and sulfate esters and is used as an ingredient in some dietary supplement products [16,17]. For the past decade, fucoidan has been extensively studied due to its varied biological activities in a number of biological systems. It has recently been reported that fucoidan possesses a wide variety of biological activities *in vitro* and *in vivo* such as anticoagulant, antithrombotic, antivirus, immunomodulatory, anti-inflammatory, antioxidant, and anticomplementary properties [17,18,19,20,21,22].

Although, accumulating evidence suggests the anticancer effects of fucoidan through the activation of apoptosis and suppression of metastasis and angiogenesis in different cancer cell types [22,23,24,25,26,27,28,29,30,31,32,33], the molecular mechanisms have not been fully clarified. Therefore, in this study, we investigated the effects of fucoidan on cell proliferation, cell cycle progression and apoptotic cell death in human urinary bladder carcinoma T24 (derived from high-grade metastatic bladder cancer) cell line, and we also attempted to clarify the possible signaling pathways involved in fucoidan-induced cell cycle arrest and apoptosis. This study is the first to determine the cell growth inhibition activity of fucoidan and examine its effect on cell cycle distribution and apoptosis in human bladder cancer cells.

## 2. Results and Discussion

### 2.1. Fucoidan-Induced Growth Inhibition is Associated with the Induction of Apoptosis in T24 Cells

**Figure 1:**
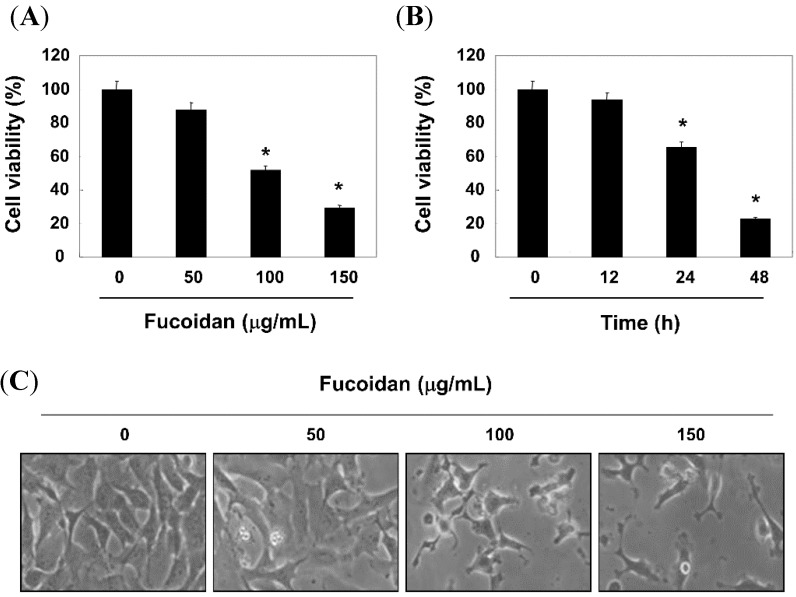
Effects of fucoidan on cell viability and morphology on T24 cells. (**A** and **B**) Cells were treated with different concentrations of fucoidan for 48 h (**A**) or 150 μg/mL fucoidan for the indicated times (**B**) Then cells were harvested to calculate the percentage of cell viability by the MTT assay. Data are presented as mean ± SD in triplicate. Significance was determined by the Student’s *t*-test (* *p* < 0.05 *vs.* untreated control); (**C**) The morphological changes of cells were imaged using an inverted microscope (original magnification, ×200).

We first tested the antiproliferative effect of fucoidan in T24 cells using a 3-(4,5-dimetylthiazol-2-yl)-2, 5-diphenyl-tetrazolium (MTT) assay. As exhibited in Figure 1A,B, the proliferative inhibitory effect of fucoidan was observed in a concentration- and time-dependent manner.

**Figure 2:**
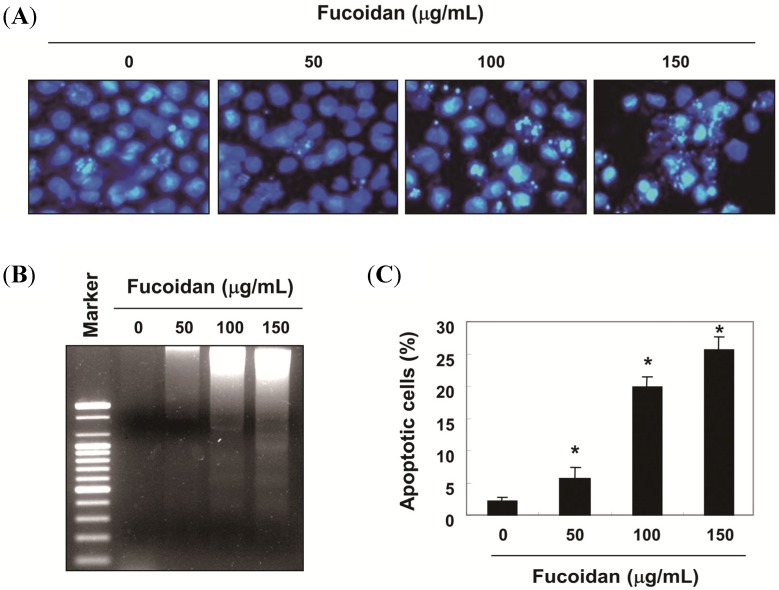
Induction of apoptotic cell death of T24 cells by fucoidan treatment. (**A**) Cells were treated with various concentrations of fucoidan for 48 h to examine the nuclear morphological changes. The cells were fixed and stained with DAPI solution. After 10 min of incubation at room temperature, stained nuclei were then observed with a fluorescent microscope (original magnification, ×400); (**B**) For the DNA fragmentation analysis, genomic DNA was extracted from cells grown under the same conditions as (A) separated by 1.0% agarose gel electrophoresis, and visualized under ultraviolet (UV) light after staining with ethidium bromide (EtBr). The marker indicates a size marker of the DNA ladder; (**C**) To quantify the degree of apoptosis induced by fucoidan, the cells were harvested to determine the percentage of annexin V positive/propidium iodide (PI) negative (apoptotic cells). The data are expressed as the mean ± SD of three independent experiments. The significance was determined by Student’s *t*-test (* *p* < 0.05 *vs.* untreated control).

Under the same conditions, fucoidan induced morphological changes such as membrane blebbing and reduced cell volume, and these effects are dose-dependent (Figure 1C). Next, nuclear morphology by 4,6-diamidino-2-phenyllindile (DAPI) staining and agarose gel electrophoresis were assessed in order to elucidate whether fucoidan inhibits cell growth through the induction of apoptosis. As shown in Figure 2A, the nuclear structure of control cells remained intact, while nuclear chromatin condensation and fragmentation, characteristic of apoptosis, was concentration-dependently increased in cells treated with fucoidan, which was associated the increased DNA fragmentation (Figure 2B). Furthermore, to measure apoptotic cell death upon fucoidan treatment, we stained cells for annexin V. As shown in Figure 2C, after treatment with 100 µg/mL and 150 µg/mL of fucoidan for 48 h, the percentages of apoptotic cells increased from approximately 2% to 20% and 26%, respectively.

### 2.2. Fucoidan Induces Cell Cycle Arrest at G1 Phase in T24 Cells

**Figure 3:**
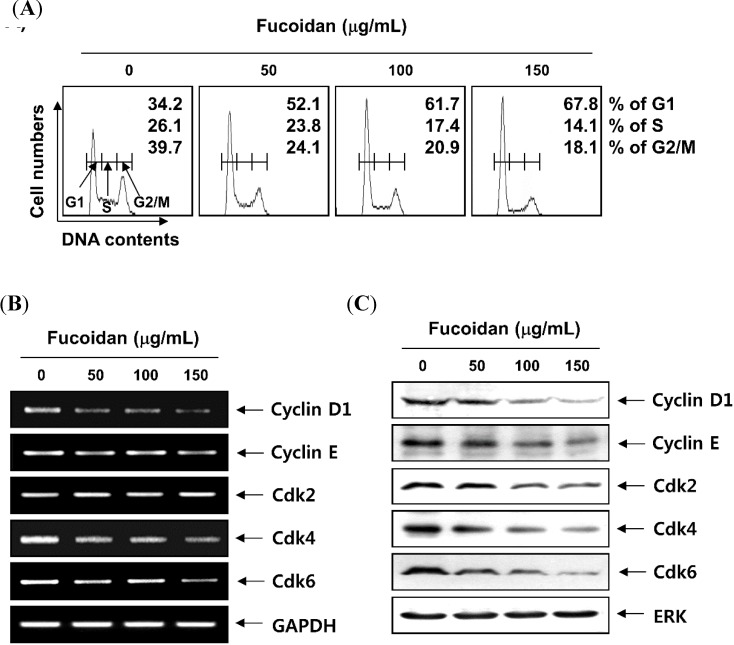
Effects of fucoidan on the cell cycle progression and expression of G1 phase-associated cyclins and cyclin-dependent kinases (Cdks) in T24 cells. (**A**) After treatment with fucoidan for 48 h, the cells were collected, fixed, and stained with PI for flow cytometry analysis. DNA content is represented on the x-axis, and the number of cells counted is represented on the y-axis. Each point represents the mean of two independent experiments; (**B**) Total RNA was isolated and reverse-transcribed. Resulting cDNAs were then subjected to PCR. The reaction products were subjected to electrophoresis in a 1.0% agarose gel and visualized by EtBr staining. Glyceraldehyde 3-phosphate dehydrogenase (GAPDH) was used as an internal control; (**C**) Cells treated with fucoidan were lysed for protein extraction. Samples were subjected to SDS-polyacrylamide gels and Western blotting for the detection of the indicated proteins. Proteins were visualized using an enhanced chemiluminescence (ECL) detection system. Extracellular-regulated kinase (ERK) was used as an internal control.

**Figure 4:**
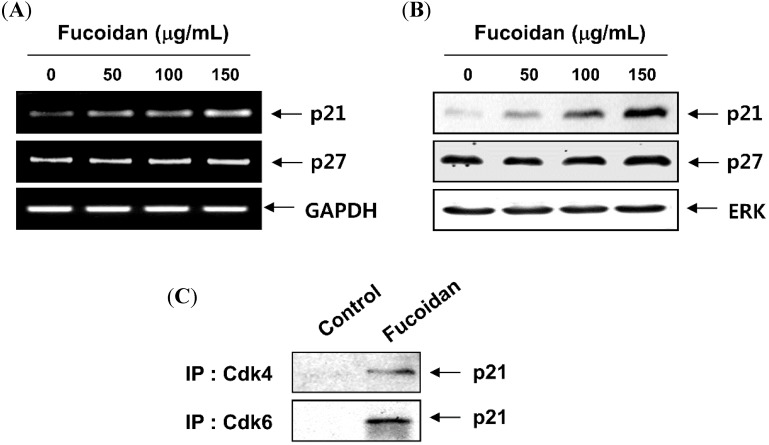
Induction of Cdk inhibitor p21 expression and its association with Cdks in response to fucoidan treatment in T24 cells. (**A**) Cells were incubated in the presence or absence of several concentrations of fucoidan for 48 h. Total RNAs were isolated and reverse-transcribed. The resulting cDNAs were then subjected to PCR with the indicated primers, and the reaction products were separated in 1.0% agarose gel and visualized by EtBr staining; (**B**) The cell lysates were separated, and equal amounts of total cell lysates were subjected to SDS-polyacrylamide gels, transferred, and probed with antibodies against p21 and p27; (**C**) After treatment with 150 µg/mL fucoidan for 48 h, total cell lysates (0.5 mg of protein) were immunoprecipitated with anti-Cdk4 or anti-Cdk6 antibody, separated on 12% SDS-polyacrylamide gels, and transferred onto nitrocellulose membrane. p21 protein levels were detected with anti-p21 antibody and the ECL detection system. (IP, immunoprecipitation).

To observe the effects of fucoidan on cell cycle progression in T24 cells, cell cycle distribution profiles were recorded by a flow cytometer. Compared with the untreated control, the number of cells in the G1 phase was increased from 34.2% to 52.1%, 61.7% and 67.8% after 48 h treatment with 50 µg/mL, 100 µg/mL, and 150 µg/mL of fucoidan, respectively (Figure 3A). To examine the molecular mechanisms of G1 arrest in T24 cells treated with fucoidan, we studied the expression of G1 phase regulators using reverse transcription-polymerase chain reaction (RT-PCR) and western blot analyses. Our data indicated that fucoidan treatment resulted in a remarkable decrease of cyclin D1, cyclin E and Cdks expression (Figure 3B,C); however, the expression of Cdk inhibitor p21 was increased at the transcriptional and translational levels, whereas the level of Cdk inhibitor p27 was not markedly altered with the same treatment (Figure 4A,B). As p53 gene is mutated in T24 cells [34], it is most likely that the induction of p21 by fucoidan is mediated through a p53-independent fashion. Because it was well known that Cdk inhibitor p21 inhibits the activity of Cdks by direct association with various cyclin/Cdk complexes, the complex formation of cyclins/Cdks/p21 is increased in cells arrested by anti-cancer agents. As shown in Figure 4C an association of p21 with Cdk4 and Cdk6 was almost undetectable by co-immunoprecipitation analysis of the untreated log phase cells. However treatment of cells with fucoidan resulted in a significant increase in the binding of these Cdks with p21 in T24 cells.

### 2.3. Fucoidan Inhibits pRB Phosphorylation and Increases the Binding of pRB and E2Fs in T24 Cells

**Figure 5:**
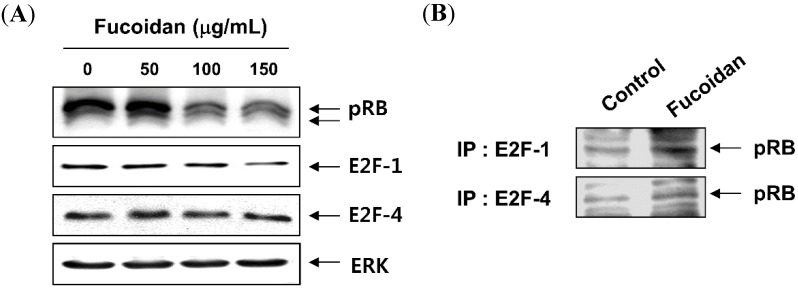
Hypophosphorylation of retinoblastoma protein (pRB) and enhanced the association of pRB and E2Fs in T24 cells after exposure to fucoidan. (**A**) Cells were treated with different concentrations of fucoidan for 48 h, and the total cell lysates were then prepared and separated by electrophoresis on an 8% or 10% SDS-polyacrylamide gels. Western blotting was then performed using anti-pRB, anti-E2F-1 and anti-E2F-4 antibodies; (**B**) Whole cell lysates (0.5 mg of protein) from control cells and cells treated with 150 th 150fucoidan were immunoprecipitated with anti-E2F-1 or anti-E2F-4 antibody. Immuno-complexes were separated by 8% SDS-polyacrylamide gel electrophoresis, transferred to a nitrocellulose membrane, and probed with anti-pRB antibody. Proteins were detected by ECL detection. (IP, immunoprecipitation).

Because fucoidan induces cell cycle arrest at G1 phase and the Rb gene product pRb is an important checkpoint in the G1 phase of the cell cycle [5,35], we next determined the kinetics between the phosphorylation of pRB and the transcription factors E2Fs. As indicated in Figure 5A, the total levels of pRB expression were decreased remarkably and changed from the hyperphosphorylated form to the hypophosphorylated form after fucoidan treatment, and these changes occurred in a time-dependent manner. Alternatively, the levels of E2F-1 and E2F-4 expression remained unchanged in T24 cells treated with fucoidan. In addition, the association of pRb and E2F-1/E2F-4 was very low in untreated log phase cells, as determined by co-immunoprecipitation analysis; however, there was a strong increase in the association of pRB and E2F-1 as well as E2F-4 in fucoidan-treated cells (Figure 5B). The results indicated that fucoidan inhibits the release of E2Fs proteins from pRB.

### 2.4. Fucoidan Induces Apoptosis through a Caspase-Dependent Cascade in T24 Cells

**Figure 6:**
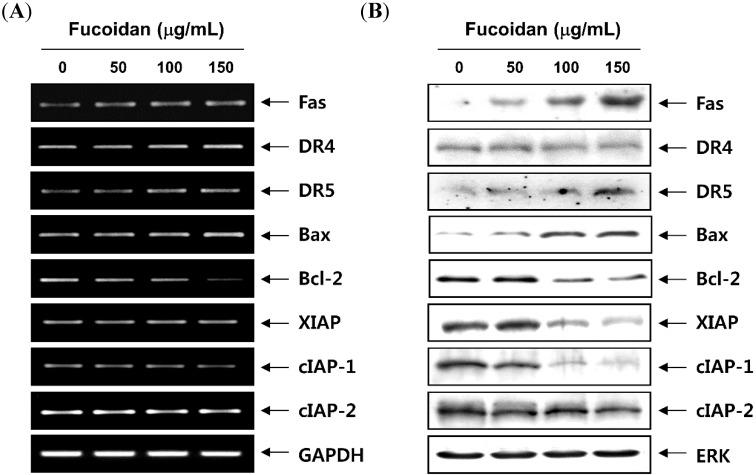
Induction of caspase-dependent apoptosis by fucoidan treatment in T24 cells. (**A** and **B**) Cells were incubated in the presence or absence of several concentrations of fucoidan for 48 h. (**A**) Cells were subjected to Western blot analysis using the indicated antibodies and an ECL detection system; (**B**). Caspase-3, -8, and -9 activity was determined with colorimetric assay kits. The data were normalized to the caspase activity within the control cells and represented as the fold of the control. Data are averages with SD from at least three independent experiments (* *p* < 0.05 *vs.* untreated control); (**C**) Cells were incubated in the presence or absence of z-VAD-fmk for 2 h before being exposed to fucoidan. After 48 h of incubation, cell viability was analyzed using an MTT assay; (**D**) Cells grown under the same conditions as (**C**) were harvested to determine the percentage of annexin V^+^/^−^ (apoptotic cells). The data are expressed as the means ± SD of triplicate samples (* *p* < 0.05 *vs.* untreated control; # *p* < 0.05 *vs.* fucoidan treated cells).

To characterize the cell death pathway triggered by fucoidan, the levels of caspases and their activities were determined by western blot analysis and a colorimetric caspase activity assay. Our results indicated that the levels of pro-forms of caspase-8, and -9, initiator caspases of extrinsic and intrinsic apoptotic pathways, respectively, were down-regulated and their activities were significantly increased in fucoidan-treated T24 cells in a concentration-dependent manner (Figure 6A,B). The increase in apoptosis was also accompanied by the activation of caspase-3, a classical downstream execution caspase, and the cleavage of the pro-form poly(ADP-ribose) polymerase (PARP) protein (116 kDa) to the inactive form (85 kDa), which has been identified as a substrate for caspase-3. To investigate if fucoidan induces apoptosis in T24 cells through a caspase-dependent pathway, cells were pretreated with a general and potent inhibitor of caspase, z-VAD-fmk, and then exposed to fucoidan for 48 h. The results indicate that z-VAD-fmk is able to protect against fucoidan-reduced cell viability and -induced apoptosis (Figure 6C,D), suggesting that the fucoidan induced apoptotic death in T24 cells through a caspase cascade-dependent pathway. 

### 2.5. Fucoidan Modulates the Levels of Apoptosis-Related Factors and MMP Values in T24 Cells

**Figure 7:**
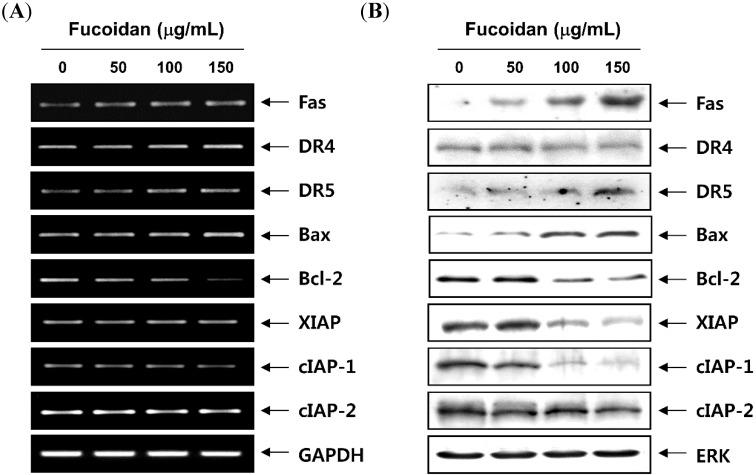
Effects of fucoidan on levels of apoptosis-related regulators in T24 cells. Cells were treated with the indicated concentrations of fucoidan for 48 h. (**A**) Total RNAs were isolated and reverse-transcribed. The resulting cDNAs were then subjected to PCR with the indicated primers and the reaction products were separated in 1.0% agarose gel and visualized by EtBr staining. GAPDH was used as an internal control; (**B**) The cell lysates were separated with SDS-polyacrylamide gel electrophoresis, and western blotting analyses were performed using the indicated antibodies and an ECL detection system.

**Figure 8:**
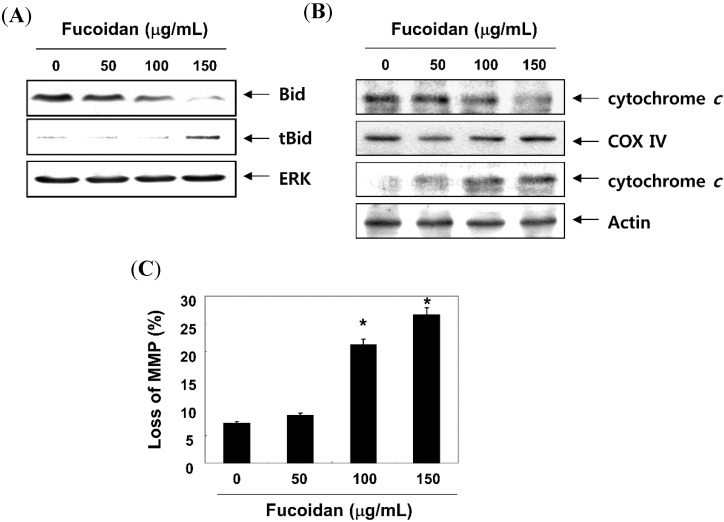
Induction of Bid truncation, cytochrome *c* translocation and mitochondrial membrane hyperpolarization by fucoidan treatment in T24 cells. The cells were treated with the indicated concentrations of fucoidan for 48 h. (**A**) Proteins on western blots were detected with anti-Bid antibody and ECL detection; (**B**) Cytosolic and mitochondrial extracts were prepared, resolved by SDS-polyacrylamide gel electrophoresis, transferred to nitrocellulose membranes, and then probed with the anti-cytochrome *c* antibodies. Cytochrome oxidase IV (COX IV) and lamin B were used as internal controls for the cytosolic and mitochondrial fractions, respectively; (**C**) The cells were stained with JC-1 for 20 min at 37 °C. The mean JC-1 fluorescence intensity was then detected using flow cytometry. The data are expressed as the means ± SD of triplicate samples (* *p* < 0.05 *vs.* untreated control).

We next determined the expression of the apoptosis-associated gene products after treatment with fucoidan to further investigate the mechanism of fucoidan-induces apoptosis in T24 cells. As shown in Figure 7, the levels of Fas and death receptor 5 (DR) mRNA and protein expression, but not DR4, were enhanced after treatment with fucoidan. The expression of the anti-apoptotic Bcl-2 proteins was suppressed; however, that of the pro-apoptotic Bax proteins increased in response to fucoidan treatment. We also examined whether the fucoidan induced apoptosis by modulating the expression of inhibitor of apoptosis protein (IAP) family members. The results indicated that fucoidan treatment caused the down-regulation of XIAP as well as the cIAP-1 and cIAP-2 expression in a concentration-dependent manner. Moreover, treatment with the fucoidan resulted in a decreased level of full-length Bid, a BH3-only protein of Bcl-2 family, and an increased appearance of a truncated Bid (tBid) band (Figure 8A), which reflected Bid cleavage and activation. Furthermore, the exposure of cells to fucoidan led to a significant increase in the release of the mitochondrial pro-apoptotic protein cytochrome *c* to the cytosol (Figure 8B). In contrast, treatment with fucoidan induced a significant decrease in cytochrome *c* in the mitochondria suggesting a direct important role of the mitochondria in the fucoidan-induced apoptosis of T24 cells. In addition, fucoidan treatment of T24 cells resulted in loss of the mitochondrial membrane potential (MMP) compared with that in untreated controls, as measured by 5,5',6,6'-tetrachloro-1,1',3,3'-tetraethylimidacarbocyanine iodide (JC-1) staining (Figure 8C), indicating that the fucoidan induced mitochondrial membrane hyperpolarization by depolarization.

### 2.6. Discussion

In term of fucoidan’s anti-cancer properties, Riou *et al.* [36] were the first to report fucoidan-induced G1 arrest of the cell cycle in cultured human non-small-cell bronchopulmonary carcinoma cells. After that, Aisa *et al.* [37] first demonstrated that fucoidan induced apoptosis in human lymphoma cells through caspase-3 activation and extracellular-regulated kinase (ERK) pathways. Since then, several studies have been done to elucidate the mechanism of fucoidan-induced cell cycle arrest and apoptosis using human cancer cells *in vitro* [25,26]; however, the molecular signaling pathways of fucoidan-induced anti-proliferative action on treating human bladder cancer cells have not yet been studied. In the present study, we employed a functional study of this agent using the T24 human urinary bladder cancer cell line. Our data showed that fucoidan could induce G1 arrest and caspase-dependent apoptosis in T24 cells. We further found that the enhanced expression of Cdk inhibitor p21 and dephosphorylation of pRB is associated with fucoidan-induced G1 arrest, and that both caspase-dependent intrinsic and extrinsic apoptotic pathways were involved in the initiation of fucoidan-triggered apoptotic cell death in T24 cells. 

In general, orderly progression through the cell cycle checkpoints involves the coordinated activation of the Cdks in the presence of associated cyclins. Passage from the G1 to the S phase is regulated by D-type cyclins and their cognate kinases Cdk4 and Cdk6, which act by phosphorylating and inactivating pRB, which consequently lead to the liberation of a transcription factor E2Fs from pRB, prior to the restriction point at which cells commit to DNA synthesis [5,35]. Successively, Cdk2 is associated with cyclin E and functions in the late G1 progression; then Cdk2 binds to cyclin A, resulting in the positive regulation of the G1 to S phase transition. The activities of cyclin/Cdk complexes are also negatively regulated by Cdk inhibitors including p21 and p27, which inhibit either Cdks kinase activity or prevent their activation by directly binding to Cdks, thereby blocking the interaction of cyclin/Cdk complexes with their substrates [3,34]. The data presented here demonstrate that in T24 cells, fucoidan led to the down-regulation of G1 regulatory cyclins and Cdks, and a marked increase in p21 expression, but not p27, associated with both transcriptional and translational levels. Furthermore, p21 was detected in anti-Cdk4 and Cdk6 immunoprecipitates from fucoidan-treated cells and not in immunoprecipitates from untreated cells. Although p21 is originally known to transcriptionally activate by tumor suppressor p53, the inactivation of p53 is a common characteristic of many tumor types while p21 is rarely inactivated in human cancer [38,39]. Because the p53 gene is mutated in T24 cells [34], it is most likely that the induction of p21 is mediated in a p53-independent fashion. Our results also show that fucoidan blocks pRB phosphorylation, without marked alteration of E2F-1 and E2F-4 proteins, and increases binding of pRB to E2Fs, consistent with the restoration of the growth-regulatory activity of pRB. These results indicate that fucoidan may induce the G1 phase arrest of T24 cells through the down-regulation of Cdks kinase activity, *via* the selective induction of p53-independent p21 and inhibition of pRB phosphorylation.

In addition to cell cycle arrest at the G1 phase by fucoidan, our data support the involvement of both intrinsic and extrinsic apoptotic signaling pathways in fucoidan-treated T24 cells, as evidenced by caspase-9 and -8 activation. In the extrinsic apoptosis pathway, Fas/Fas legend (FasL) system is a key signaling transduction pathway. Ligation of Fas by its natural ligand FasL induces receptor oligomerization and the formation of death-inducing signaling complex, followed by the activation of caspase-8, then further activating a series of caspase cascades resulting in cell apoptotic death [11,40]. In our results, indicating an increased Fas protein level, the cleavage of Bid protein by fucoidan in T24 cells suggests that processing/activation of pro-caspase-8 drives the caspase cascade with the requirement of the loop amplification from the mitochondria. Supporting this hypothesis, fucoidan decreased the level of MMP values and promoted the release of cytochrome *c* from mitochondria, indicating that fucoidan caused a collapse of electrochemical gradient across the mitochondrial membrane, and subsequently increased the activity of caspase-9. Fucoidan also activated effector caspase-3, followed by the cleavage of PARP, a nuclear enzyme involved in DNA repair in response to various stresses. The intrinsic pathway is also controlled by the Bcl-2 family members, which play important regulatory roles in apoptosis, by either inhibiting or promoting apoptosis. Heterodimerization between the pro- (such as Bcl-2 and Bcl-xL) and anti-apoptotic proteins (such as Bax and Bad) of this family and the balance of Bcl-2 family members may determine the susceptibility to a given apoptotic stimulus and cell fate [10,41]. In this study, fucoidan-induced apoptosis was concurrent with the increasing of Bax and decreasing of Bcl-2 resulting in an increase in the ratio of Bax/Bcl-2, further suggesting fucoidan induced apoptosis by evoking the intrinsic apoptosis pathway. Apoptosis may be inhibited by various proteins, including members of the IAP family, which are largely overexpressed by most tumors. IAP family proteins promote tumor cell survival due to direct inhibition by binding to several caspases after a wide variety of apoptotic stimuli [42,43]. Our results revealed that fucoidan-induced apoptosis was related to the down-regulation of IAP family numbers such as XIAP, cIAP-1 and cIAP-2 (Figure 7), in association with the activation of caspases. However, under the same experimental conditions, significant protection of fucoidan-induced growth inhibition and apoptosis was observed following pretreatment with a pan caspase inhibitor, z-VED-fmk (Figure 6). Although further studies are needed, our results indicate that the caspase-dependent intrinsic and extrinsic pathways contribute, at least in part, to fucoidan-induced apoptosis in T24 cells. 

## 3. Experimental

### 3.1. Materials

Fucoidan, MTT, PI, and DAPI were purchased from Sigma-Aldrich (St. Louis, MO, USA). Fetal bovine serum (FBS) and caspase activity assay kits were obtained from GIBCO-BRL (Gaithersburg, MD, USA) and R&D Systems (Minneapolis, MN, USA), respectively. The pan-caspase inhibitor, z-VAD-fmk, and JC-1 were purchased from Calbiochem (San Diego, CA, USA). DNA staining and ECL kits were purchased from Becton Dickinson (San Jose, CA, USA) and Amersham Corp (Arlington Heights, IL, USA), respectively. Antibodies were purchased from Santa Cruz Biotechnology (Santa Cruz, CA, USA), Calbiochem, and Cell Signaling Technology (Danvers, MA, USA). The peroxidase-labeled donkey anti-rabbit immunoglobulin and peroxidase-labeled sheep anti-mouse immunoglobulin were purchased from Amersham Corp. All other chemicals were purchased from Sigma-Aldrich.

### 3.2. Cell Culture, MTT Assay and Determinations of Cell Morphology

The human urinary bladder cancer T24 cell line, which was derived from an undifferentiated grade III carcinoma [44], was obtained from the American Type Culture Collection (Manassas, VA, USA). The cells were cultured in RPMI 1640 medium supplemented with 10% FBS and 1% penicillin-streptomycin at 37 °C in a humid environment containing 5% CO_2_. Fucoidan was dissolved in phosphate buffered saline (PBS) as a stock solution at a 200 mg/mL concentration, and the stock solution was then diluted with the medium to the desired concentration prior to use. The MTT reduction assay was used to determine cell viability. In brief, cells were treated with various concentrations of fucoidan for 48 h or 150 µg/mL of fucoidan for the indicated time periods. Following treatment, the medium was removed and the cells were incubated with 0.5 mg/mL of MTT solution. After incubation for 2 h at 37 °C and 5% CO_2_, the supernatant was removed and formation of formazan was measured at 540 nm with a microplate reader. In the end of incubation, cells from each well were examined and photographed under a phase-contrast microscope (Carl Zeiss, Oberkochen, Germany).

### 3.3. Nuclear Staining with DAPI

After treating the cells with fucoidan, they were harvested, washed in ice-cold PBS, and fixed with 3.7% paraformaldehyde in PBS for 10 min at room temperature. The fixed cells were washed with PBS and stained with a DAPI solution (2.5 µg/mL) for 10 min at room temperature. Changes in the nuclear morphology of the cells were analyzed using a fluorescence microscope (Carl Zeiss).

### 3.4. DNA Fragmentation Assay

After fucoidan treatment, the cells were lysed in a buffer containing 10 mM of Tris-HCl, pH 7.4, 150 mM of NaCl, 5 mM of EDTA, and 0.5% Triton X-100 for 1 h at room temperature. The lysates were vortexed and cleared by centrifugation at 15,000 rpm for 10 min at 4 °C. The DNA in the supernatant was extracted using a 25:24:1 (*v/v/v*) equal volume of neutral phenol:chloroform:isoamyl alcohol. To assay the DNA fragmentation pattern, samples were loaded onto 1.0% agarose gel containing 0.1 µg/mL of EtBr, and electrophoresis was carried out.

### 3.5. Measurement of Apoptosis by Flow Cytometry

After incubation with fucoidan, the cells were washed twice with PBS and resuspended in an annexin-V binding buffer containing 10 mM of HEPES/NaOH (pH 7.4), 140 mM of NaCl, and 2.5 mM of CaCl_2_. Aliquots of the cells were incubated with annexin-V fluorescein isothiocyanate (FITC, R&D Systems), mixed, and incubated for 15 min at room temperature in the dark. The cells were washed with PBS and PI at a concentration of 5 µg/mL was added to distinguish the necrotic cells. Fluorescent signals were measured using a flow cytometer (FACS Calibur; Becton Dickinson) [45].

### 3.6. Cell Cycle Analysis

At the end of the treatment, the cells were collected and washed twice with PBS. To determine cell cycle distribution, the cell pellet was resuspended in 70% (*v/v*) ethanol and stored at 4 °C overnight. The cells were then pelleted and resuspended in 1 ml of PBS containing 20 µg/mL of RNase A. After incubation with RNase A for 30 min, the cells were stained with PI solution and flow cytometry analysis was carried out using a flow cytometer. 

### 3.7. RNA Extraction and RT-PCR

Total RNAs were isolated using an RNeasy mini kit (Qiagen, La Jolla, CA, USA) and primed with random hexamers to synthesize complementary DNA following the manufacturer’s instructions (Amersham). PCR was performed in a Mastercycler (Eppendorf, Hamburg, Germany) with the indicated primers. The conditions for PCR reactions were 1X (94 °C for 3 min), 35X (94 °C for 45 s; 58 °C for 45 s; and 72 °C for 1 min), and 1X (72 °C for 10 min). The amplification products obtained by PCR were electrophoretically separated on a 1.0% agarose gel and visualized by EtBr staining.

### 3.8. Western Blot Analysis

The total cellular proteins were extracted with lysis buffer (20 mM of sucrose, 1 mM of EDTA, 20 µM of Tris-HCl, pH 7.2, 1 mM of DTT, 10 mM of KCl, 1.5 mM of MgCl_2_ and 5 µg/mL of aprotinin) for 30 min. The mitochondrial and cytosolic fractions were isolated using a mitochondrial fractionation kit (Activemotif, Carlsbad, CA, USA) according to the manufacturer’s instructions. The protein concentration was measured using a Bio-Rad protein assay (Bio-Rad Lab., Hercules, CA, USA) according to the manufacturer’s instructions. Cell extracts containing 30~50 µg of proteins were separated on SDS-polyacrylamide gels and transferred to nitrocellulose membranes (Schleicher & Schuell, Keene, NH, USA). After 2 h blocking with 5% (*w/v*) nonfat milk in TBST (1.5 M of NaCl, 20 mM of Tris–Cl, 0.05% (*v/v*) Tween-20, pH 7.4), the membranes were incubated overnight at 4 °C with the desired antibodies. The blots were then washed with TBST 2 h prior to incubation at room temperature with peroxidase conjugated secondary antibodies. Proteins were visualized by using ECL detection method followed by film exposure.

### 3.9. Co-Immunoprecipitation Assay

The binding activity of proteins was determined by co-immunoprecipitation assay. For this study, total cell lysates were incubated with the desired antibodies for 1 h at 4 °C and the immuno-complex was collected on protein A-Sepharose beads for 1 h and washed 5 times with lysis buffer prior to boiling them in SDS sample buffer. Immunoprecipitated proteins were separated on SDS-polyacrylamide gels and transferred to nitrocellulose membranes for Western blot analysis. 

### 3.10. In Vitro Caspase Activity Assay

The caspase activities were determined by colorimetric assays using caspase-3, -8, and -9 activation kits according to the manufacturer’s protocol. The kits utilize synthetic tetrapeptides labeled with pnitroaniline (pNA). Briefly, the cells were lysed in the supplied lysis buffer for 30 min. The supernatants were collected and incubated at 37 °C with the reaction buffer, which contained dithiothreitol and the substrates Asp-Glu-Val-Asp (DEVD)-p-nitroaniline (pNA) for caspase-3, Ile-Glu-Thr-Asp (IETD)-pNA for caspase-8, and Leu-Glu-His-Asp (LEHD)-pNA for caspase-9, respectively. The caspase activity was determined spectrophotometrically by measuring changes in absorbance at 405 nm using the ELISA reader.

### 3.11. Assay of MMP (ΔΨm)

To determine the level of MMP (*ΔΨm*), the cells were harvested at the end of incubation, and the cell pellets were resuspended in PBS and incubated with 10 μM JC-1 at dark room for 20 min at 37 °C. Subsequently, the cells were washed once with cold PBS, suspended, and analyzed immediately by a flow cytometry. 

### 3.12. Statistical Analyses

The statistical analysis was performed with the SPSS17.0 software package (SPSS, Inc., Chicago, IL, USA). Values were expressed as the means ± SD of the mean. All experiments were repeated at least three times. The significance of the differences between the controls and each experimental group was analyzed with an unpaired Student’s *t*-test, and *p* < 0.05 was considered statistically significant.

## 4. Conclusions

Based on these observations, we suggest that fucoidan decreases the viability of T24 cells through the cell cycle arrest in the G1 phase and the induction of apoptosis. Fucoidan-induced G1 arrest was associated with decreasing pRB phosphorylation and enhanced p53-independent induction of the Cdk inhibitor, p21. Fucoidan induced apoptosis in T24 cells *via* the extrinsic and intrinsic pathways through the upregulation of Fas and the loss of MMP, then led to cytochrome *c* release from mitochondria and promoted the activation of caspases, leading to the apoptosis of T24 cells, which was mediated by modulation in Bcl-2 and IAP family members. Although, these phenomena suggest that fucoidan and its related compounds may have significant potential as targets for cancer treatment, *in vivo* studies using preclinical invasive models are needed to fully establish the potential of fucoidan as a chemopreventive and therapeutic agent in bladder cancer.
